# Microbial 2-butanol production with *Lactobacillus diolivorans*

**DOI:** 10.1186/s13068-019-1594-5

**Published:** 2019-11-06

**Authors:** Hannes Russmayer, Hans Marx, Michael Sauer

**Affiliations:** 1 CD Laboratory for Biotechnology of Glycerol, Muthgasse 18, 1190 Vienna, Austria; 2 Department of Biotechnology, Institute of Microbiology and Microbial Biotechnology, BOKU-VIBT University of Natural Resources and Life Sciences, Vienna, Muthgasse 18, 1190 Vienna, Austria; 3 ACIB GmbH, Muthgasse 11, 1190 Vienna, Austria

**Keywords:** Industrial microbiology, Metabolic engineering, 2,3-Butanediol, 2-Butanone, *Serratia marcescens*

## Abstract

**Background:**

Biobutanol has great potential as biofuel of the future. However, only a few organisms have the natural ability to produce butanol. Amongst them, *Clostridium* spp. are the most efficient producers. The high toxicity of biobutanol constitutes one of the bottlenecks within the biobutanol production process which often suffers from low final butanol concentrations and yields. Butanol tolerance is a key driver for process optimisation and, therefore, in the search for alternative butanol production hosts. Many *Lactobacillus* species show a remarkable tolerance to solvents and some *Lactobacillus* spp. are known to naturally produce 2-butanol from meso-2,3-butanediol (meso-2,3-BTD) during anaerobic sugar fermentations. *Lactobacillus diolivorans* showed already to be highly efficient in the production of other bulk chemicals using a simple two-step metabolic pathway. Exactly, the same pathway enables this cell factory for 2-butanol production.

**Results:**

Due to the inability of *L. diolivorans* to produce meso-2,3-BTD, a two-step cultivation processes with *Serratia marcescens* has been developed. *S. marcescens* is a very efficient producer of meso-2,3-BTD from glucose. The process yielded a butanol concentration of 10 g/L relying on wild-type bacterial strains. A further improvement of the maximum butanol titer was achieved using an engineered *L. diolivorans* strain overexpressing the endogenous alcohol dehydrogenase pduQ. The two-step cultivation process based on the engineered strain led to a maximum 2-butanol titer of 13.4 g/L, which is an increase of 34%.

**Conclusion:**

In this study, *L. diolivorans* is for the first time described as a good natural producer for 2-butanol from meso-2,3-butanediol. Through the application of a two-step cultivation process with *S. marcescens,* 2-butanol can be produced from glucose in a one-vessel, two-step microbial process.

## Background

Fossil oil constitutes the primary energy carrier worldwide, whereof around 80% are consumed within the transport sector. Environmental concerns and limited resources stimulate the quest for renewable alternatives for fuel production. Biodiesel and bioethanol are the most frequently used biofuels for diesel and gasoline engines today. In the last decade, biobutanol is becoming more and more important as alternative to the commonly used biofuels. Several advantages of biobutanol, such as a higher energy content, usability in pure form or the ability to blend it in every concentration with gasoline, favour butanol over other biofuels [1]. Butanol is a C4-alcohol. Four different isomers exist, of which 1-butanol, 2-butanol and isobutanol are the most promising candidates for biofuel production.

The largest share of the global biobutanol market has 1-butanol. Traditionally, 1-butanol is produced by ABE (acetone–butanol–ethanol) fermentation of solventogenic *Clostridia*, mainly *C. acetobutylicum* and *C. beijerinckii.* As 1-butanol is a toxic metabolite, the titers and yields are rather low. Usually, titers for wild-type strains on glucose as carbon source are around 15–20 g/L and yields are around 0.20 g/g [2]. The high toxicity of butanol constitutes a major bottleneck for high-level production of butanol with *Clostridium* spp. Metabolic engineering of *C. acetobutylicum* to increase 1-butanol production focused mainly on improving butanol tolerance and avoiding by-product formation [2]. One of the highest titers for metabolically engineered *C. acetobutylicum* strains was around 20 g/L [3, 4]. Only via process engineering, in situ removing of 1-butanol from the broth by gas stripping or liquid–liquid extraction, titers could be further improved. For a 1-butanol fermentation process using a long-term-adapted mutant of *C. acetobutylicum* JB200 in combination with in situ removal of 1-butanol, a final titer of 118 g/L for the overall fermentation was reached [5].

Isobutanol is also a promising candidate for biobutanol production, because of its lower toxicity for microbial cells. On academic level, isobutanol is mainly produced by metabolically engineered *Escherichia coli* and *Saccharomyces cerevisiae* via introduction of genes of the keto acid pathway. Engineered *E. coli* strains reached up to 22 g/L and further process engineering led to 50 g/L of isobutanol [6]. Several companies, such as Gevo Inc. (http://www.gevo.com) are involved in scale-up of the fermentation process to an industrial level.

Up to now, the third isomer 2-butanol lives in the shadow of the other two isomers, but is with its comparable higher octane number and lower toxicity to 1-butanol of considerable interest for the biofuel industry.

2-Butanol is known to be produced by some *Lactobacillus* spp. through reduction of 2,3-butanediol (2,3-BTD) during anaerobic sugar fermentation. Two consecutive enzymatic steps carry out this reduction. The first step is the dehydration of meso-2,3-BTD to 2-butanone by a vitamin B_12_-dependent glycerol dehydratase. In the second step, 2-butanone is reduced to 2-butanol by an alcohol dehydrogenase, which accepts secondary alcohols. Both enzymatic steps are located within bacterial micro-compartments (BMC), organelle-like proteinaceous structures.

**Fig. 1:**
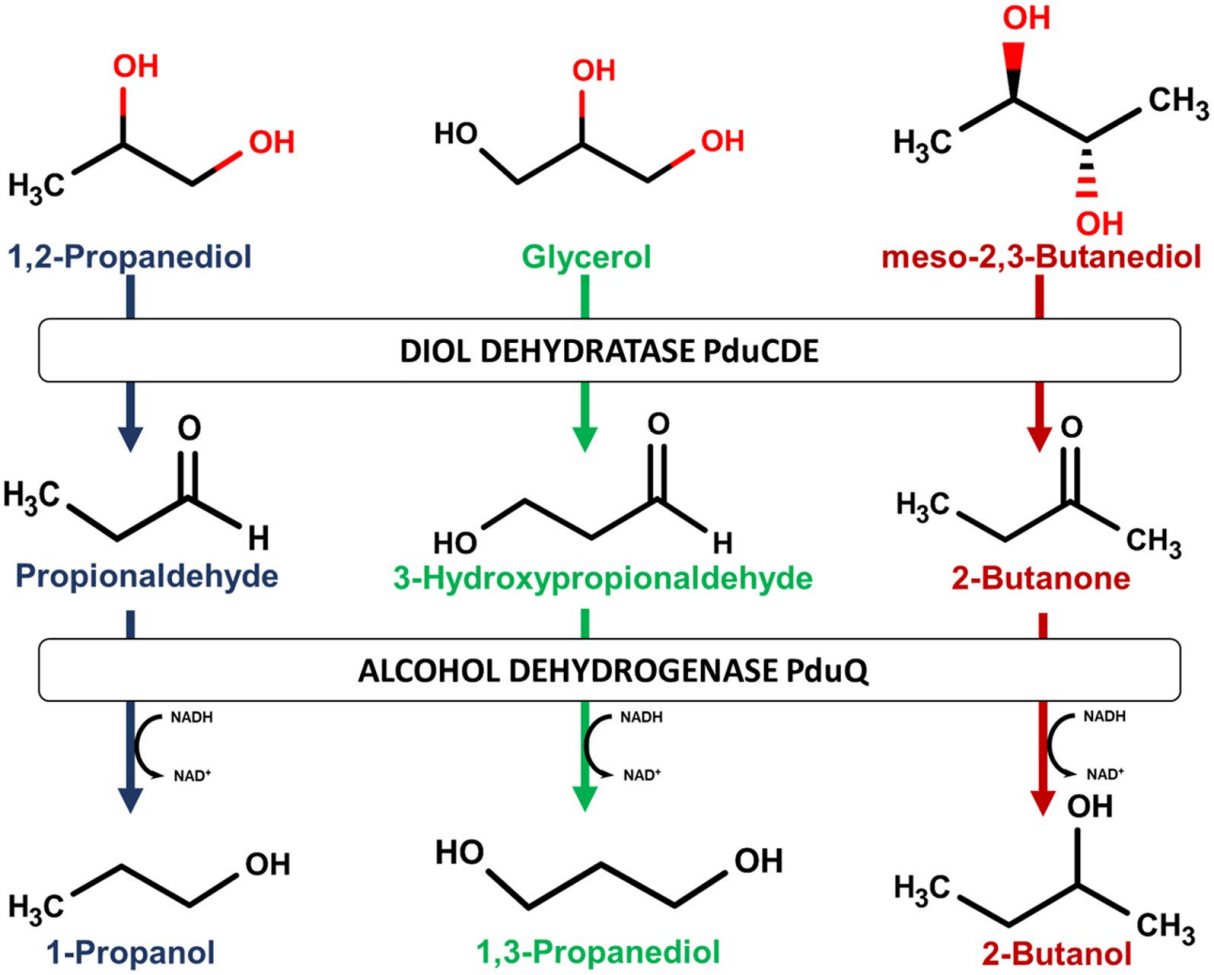
Metabolic pathways for the formation of 1-propanol, 1,3-propanediol and 2-butanol. The two major enzymes present in *L. diolivorans*, catalysing these reactions, are a vitamin B_12_-dependent diol dehydratase (PduCDE) and an alcohol dehydrogenase (pduQ)

Typically, BMCs found in *Lactobacillus* spp. are assigned to the group of propanediol utilisation (pdu) micro-compartments, due to their natural metabolic function for degradation of 1,2-propanediol. Interestingly, the same metabolic pathway located in the pdu micro-compartment is able to convert different substrates with similar chemical structures, bearing at least one vicinal diol (Fig. 1).

A *Lactobacillus* spp. known to use this metabolic pathway in a very efficient way is *Lactobacillus diolivorans*. *L. diolivorans* is able to produce up to 92 g/L of 1,3-propanediol (1,3-PDO) in fed-batch cultivations [7]. Additionally, this cell factory is able to produce up to 35 g/L of 3-hydroxypropionaldehyde (3-HPA), the intermediate product of the metabolic pathway (Fig. 1). Having this efficient and metabolically well-balanced pathway, *L. diolivorans* is also a potential candidate for 2-butanol production from 2,3-BTD. 2,3-BTD has two stereoactive centres and therefore, three different isomers are found in nature, which are the optical active R- and S-form and the inactive meso-form. All three isomers are produced by microbial strains found in nature. However, the ratio between the different isomers varies amongst the bacteria capable of producing 2,3-BTD [8]. 2,3-BTD is usually formed via the pyruvate-diacetyl/acetoin pathway. This pathway is found in lactic acid bacteria and glucose and citrate fermenting microorganisms, belonging to the genus *Klebsiella*, *Enterobacter*, *Bacillus*, and *Serratia* [9]. The mentioned pathway consists of 3 enzymatic steps starting by the condensation of two pyruvate molecules to alpha-acetolactate with the help of alpha-acetolactate synthase. In the next step, alpha-acetolactate decarboxylase decarboxylates alpha-acetolactate to acetoin. Acetoin is then used as a precursor for the production of 2,3-BTD catalysed by different acetoin reductases (or 2,3-BTD dehydrogenases). The ratio between the different isoforms for 2,3-BTD is dependent on the expressed dehydrogenase enzymes.

A variety of lactic acid bacteria (such as *Lactococcus lactis*, *Lactobacillus plantarum* and *L. brevis*) are able to produce 2,3-BTD. However, the 2,3-BTD titers obtained are very low and a racemic mixture of 2,3-BTD is produced. For high-level production of 2-butanol, a higher titer of meso-2,3-BTD and a higher stereospecificity for the meso-form are needed, because known dehydratase enzymes are usually specific for one racemic form. The benchmark for 2,3-BTD production is *Klebsiella pneumoniae* and *Serratia marcescens* reaching titers up 75.2 g/L in fed-batch cultivations with glucose as carbon source [10]. Additionally, *S. marcescens* has the advantage of producing only meso-2,3-BTD, which seems ideal for the production of 2-butanol using *L. diolivorans* [9, 11].

In this study, we investigated the potential of *L. diolivorans* for 2-butanol production. This lactic acid bacterium has several characteristics, which make this organism an interesting production host. Lactic acid bacteria are generally known to have a high tolerance to several stress conditions, such as high concentrations of acids or alcohols [12]. For example, lactic acid bacteria tolerate up to 3% butanol in the cultivation medium; whereas, *Clostridium* spp., the benchmark for 1-butanol production, typically only tolerate around 2%.

Furthermore, this organism already proved to be an efficient cell factory for metabolite production [7, 13]. The meso-2,3-BTD production by lactic acid bacteria is not high enough to realise the full potential for 2-butanol production of *L. diolivorans*. Therefore, a two-step co-cultivation process with *S. marcescens* in batch mode was developed. *S. marcescens* was selected for production of meso-2,3-BTD from glucose [9, 11]. In the first step of the process, *S. marcescens* was used to produce stereo specifically meso-2,3-BTD from glucose followed by heat inactivation of *S. marcescens*. The accumulated meso-2,3-BTD is then converted during anaerobic fermentation with glucose into 2-butanol by *L. diolivorans*.

## Results

### Butanol tolerance of *L. diolivorans*

**Fig. 2:**
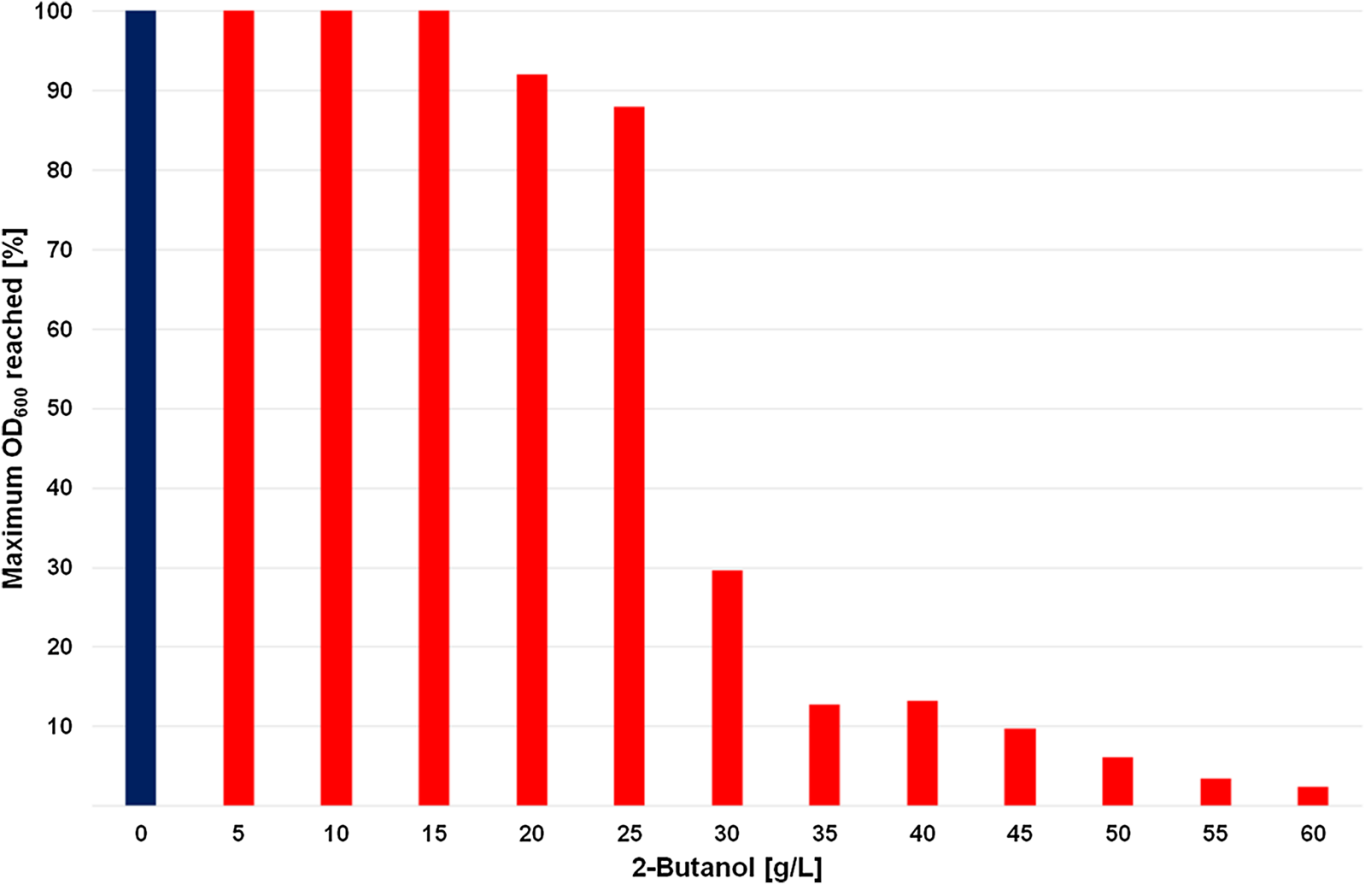
Maximum OD_600_ of *L. diolivorans* on medium with increasing butanol concentrations relative to medium without butanol

Butanol is highly toxic for most microorganisms also at low concentrations (< 15 g/L for 1-butanol). Increasing concentrations of butanol affect the membrane fluidity and impair membrane transport functions, which leads to uncontrolled efflux of intracellular components, such as proteins and metabolites [14]. Lactic acid bacteria (such as *L. brevis*) already showed to have a higher butanol tolerance than most other organism [12, 15]. Therefore, it is of interest to determine the butanol tolerance of *L. diolivorans*. The tested *L. diolivorans* wild-type strain showed a butanol tolerance up to 25 g/L 2-butanol, where it still reached 88% of the maximum OD_600_ after 72 h of incubation (Fig. 2). The maximum OD_600_ refers to the optical density reached in the control media, where no 2-butanol was added (MRS medium + 0% 2-butanol). A drastic decrease in growth was observed at 30 g/L 2-butanol, where only 30% of the maximum OD_600_ was reached, but the (non-adapted) organisms were still able to grow. The obtained results show that *L. diolivorans* has a high tolerance to solvents and is an ideal microbial cell factory for 2-butanol production. Furthermore, it was shown that *Lactobacillus* spp. have a comparable tolerance to the more toxic 1-butanol than Clostridium strains, which are the benchmark for butanol production. Wild-type *Clostridium* spp. (such as *C. acetobutylicum*) usually tolerate butanol concentrations up to 20 g/L [15, 16]. Only engineered or mutagenized *Clostridium* strains can tolerate a bit higher concentrations of butanol. Other organisms engineered for butanol production (*Escherichia coli*, *Saccharomyces cerevisiae*) exhibit mostly a lower butanol tolerance than *Clostridium* spp.

### Microbial meso-2,3-butanediol production with *S. marcescens*

Glycerol dehydratases found in lactic acid bacteria are highly stereospecific. Some have been described to only convert the meso-form of 2,3-BTD further into 2-butanone [17]. To determine if the glycerol dehydratase of *L. diolivorans* shows the same specificity, anaerobic batch cultivations with a racemic mixture of 2,3-BTD were performed. GC–MS analysis of the supernatant showed that during the anaerobic batch fermentation exclusively the meso-form of 2,3-BTD was converted into 2-butanol and the other two isomers (R-, S-form) remained untouched (data not shown). Therefore, it became clear that a process for 2,3-BTD should also exclusively yield the meso-form to aim for high overall efficiency.

**Fig. 3:**
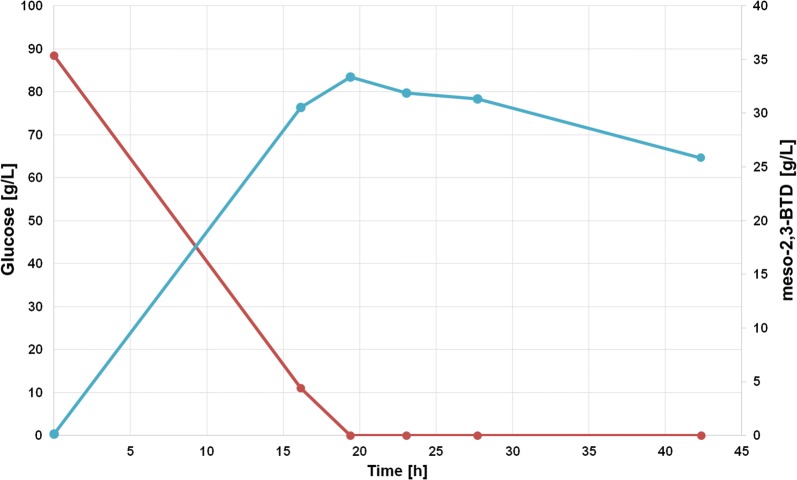
Batch cultivation of *S. marcescens* on glucose for meso-2,3-butanediol production. Glucose, closed red circles; meso-2,3-BTD, closed turquoise circles

Our efforts concentrated on the enantiospecific production of meso-2,3-BTD from glucose with *S. marcescens*. To evaluate the potential of *S. marcescens* DSMZ 14187 for meso-2,3-BTD production, batch cultivations with glucose as sole carbon source were conducted (Fig. 3). For all performed cultivations, the highest titers of meso-2,3-butanediol were reached at around 19 h with 35–39.4 g/L and a conversion yield between 0.38 and 0.44 g/g, which is close to the theoretical maximum of 0.50 g/g (Fig. 3). Our results correlate well with reported batch cultivations reaching a final titer of 42.5 g/L [18], showing *S. marcescens* DSMZ 14187 as a robust and very efficient producer of meso-2,3-BTD. Further, HPLC analysis showed that after glucose depletion, the produced meso-2,3-BTD was degraded most likely to its precursor acetoin. Therefore, it is of high importance to stop the fermentation process before glucose depletion to reach the maximum titer of meso-2,3-BTD. A part of meso-2,3-BTD the byproducts lactate, ethanol and CO_2_ were observed during the batch process on glucose. Acetate was co-utilised by *S. marcescens* as carbon source.

### Production of 2-butanol from meso-2,3-butanediol with *L. diolivorans*

**Fig. 4:**
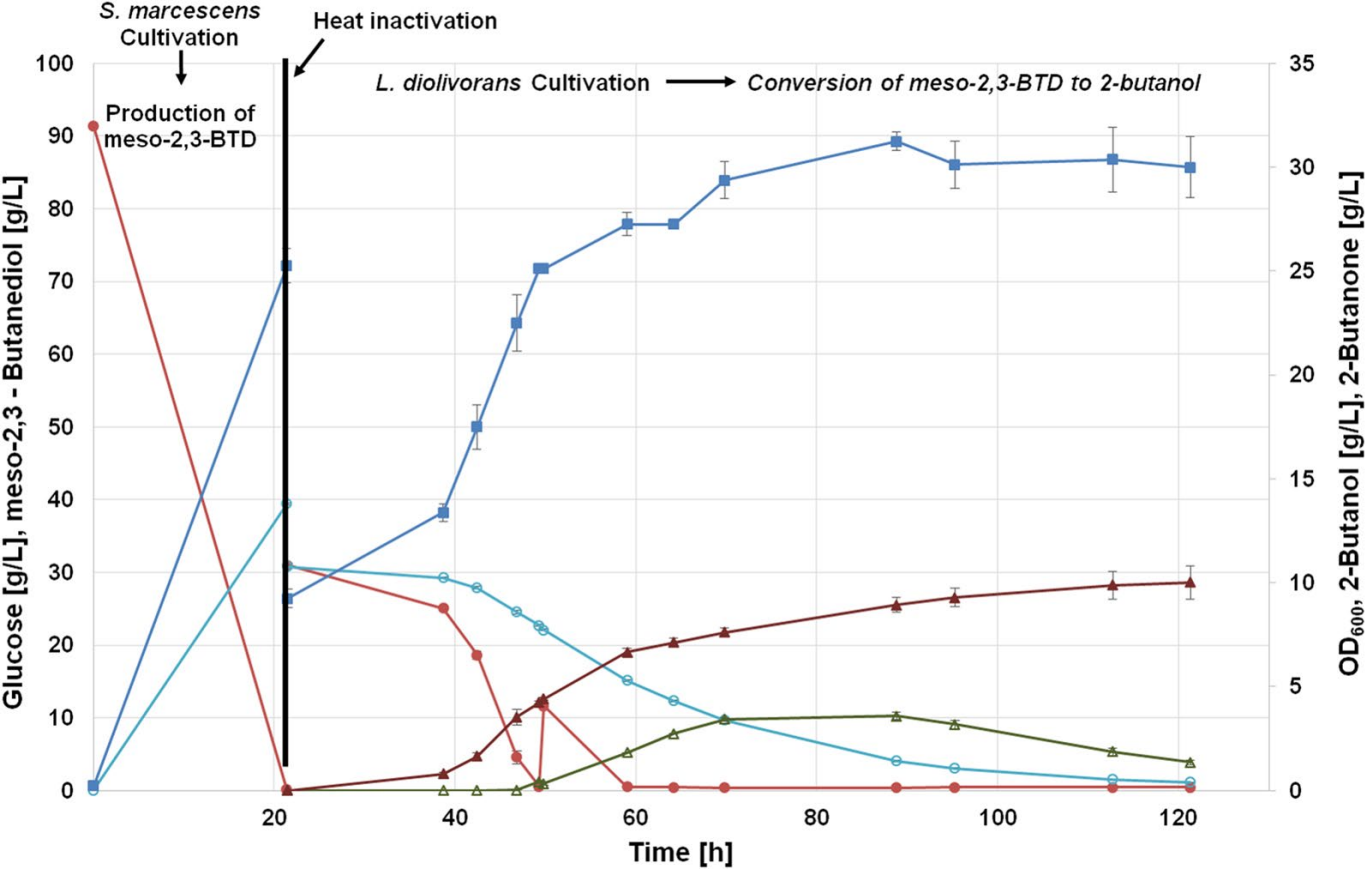
Two-step cultivation process in batch mode with wild-type *L. diolivorans.* Glucose, closed red circles; meso-2,3-BTD, open turquoise circles; OD_600_, closed blue squares; 2-butanone, opened green triangle; 2-butanol, closed brown triangle. Error bars represent the standard deviation of three independent replicates

*Serratia marcescens* was heat inactivated, when the maximum titre of around 39 g/L of meso-2,3-BTD was reached (Fig. 4). Heat inactivation was essential to prevent *S. marcescens* to further convert glucose, which is required as carbon source for the added lactic acid bacteria in the second step of the cultivation. To allow the proper growth of *L. diolivorans*, 5 times concentrated MRS plus glucose was added to the bioreactor containing the heat inactivated first-step culture. The concentration for glucose and meso-2,3-BTD at the beginning of the second phase were both 30 g/L. *L. diolivorans*, as a heterofermentative lactic acid bacterium, metabolises glucose to its typical fermentation products lactic acid, ethanol and CO_2_. In addition, acetate, 2-butanone and 2-butanol accumulated during the batch phase (Table 1). Acetate is a product accumulated on glucose when further electron acceptors such as glycerol or 2,3-BTD as in this case are present [13]. The maximum 2-butanol titre reached was 10.0 g/L after 121 h, which is the highest titer reported for microbial 2-butanol production (Fig. 4). Comparable initial concentration of glucose and glycerol, instead of meso-2,3-BTD, yielded 23.8 g/L of 1,3-PDO after 147 h [13]. Interestingly, looking at the final product titers and yields, the efficiency of the metabolic pathway for reduction of either meso-2,3-BTD or glycerol to their respective products is different. During glucose/meso-2,3-BTD fermentation, the accumulation of the intermediate product 2–butanone is observed. The formation of 2-butanone started at around 50 h of cultivation and reached the highest concentration with 3.6 g/L after 88 h (Fig. 4). However, the produced 2-butanone was depleted at the end of the fermentation. 2-butanone was either converted to 2-butanol or evaporated from the fermentation broth due to nitrogen gassing of the reactor. The accumulation of 2-butanone points to a bottleneck at the second step of the metabolic pathway, which is catalysed by an alcohol dehydrogenase. For glucose/glycerol co-fermentations, no accumulation of the intermediate 3-HPA was observed, showing that this pathway is well balanced for glycerol conversion [19]. 2-butanone accumulation can be explained by the fact that reduction to 2-butanol requires a secondary alcohol dehydrogenase. We speculate that the natural alcohol dehydrogenase is very efficient for primary alcohols but less efficient for secondary alcohols.

**Table 1:** Summary of fermentation productions after 120 h of fermentation

	Glucose (g/L)	Lactate (g/L)	Acetate (g/L)	meso23-BTD (g/L)	Ethanol (g/L)	2-Butanone (g/L)	2-Butanol (g/L)
Wild type	0.00	19.87	19.04	1.12	9.96	1.35	10.01
pduQ overexpression strain	0.00	18.91	23.31	0.28	9.99	0.60	13.41

More glucose was added to the reactor at the batch end (around 28 h) to ensure sufficient supply with NADH to fully convert meso-2,3-BTD to 2-butanol. The glucose pulse was 10 ml of a 50% glucose solution and final glucose concentration after the pulse was 11 g/L (Fig. 4). The conversion yield for 2-butanol from meso-2,3-BTD was 0,37 g/g and the overall yield for production of 2-butanol on glucose was 0.06 g/g.

### Overexpression of the endogenous 1,3-propanediol oxidoreductase (pduQ) increases 2-butanol formation

**Fig. 5:**
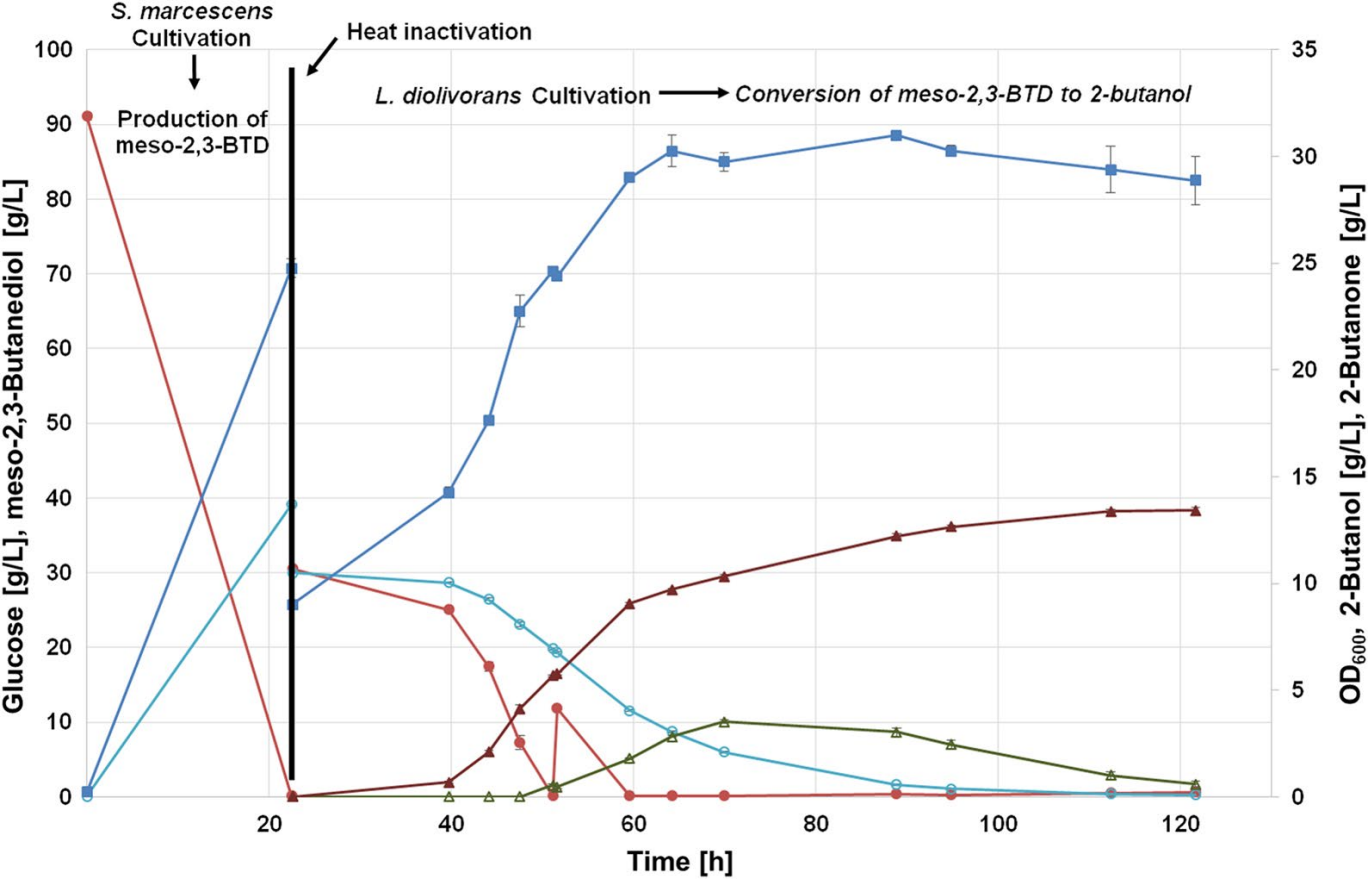
Two-step cultivation process in batch mode with *L. diolivorans* overexpressing pduQ. Glucose, closed red circles; meso-2,3-BTD, open turquoise circles; OD_600_, closed blue squares; 2-butanone, opened green triangle; 2-butanol, closed brown triangle. Error bars represent the standard deviation of three independent replicates

Batch cultivations of wild-type *L. diolivorans* indicated that the bottleneck for 2-butanol production is the reduction of 2-butanone to 2-butanol. To enhance this conversion, the overexpression of the endogenous 1,3-propanediol oxidoreductase (pduQ) was our first choice. Its promiscuous activity leads to the formation of 2-butanol from 2-butanone, as observed in the wild-type strain. The *L. diolivorans* strain overexpressing pduQ was cultivated with *S. marcescens* following the same process design (Fig. 5). The pduQ overexpression strain showed already in a previous study an increased 1,3-propanediol production from glycerol in batch cultivations, which indicates that overexpressing of pduQ positively influences product formation. Furthermore, the authors showed that pduQ enzyme activity was twice as high as in the wild-type strain [20].

**Fig. 6:**
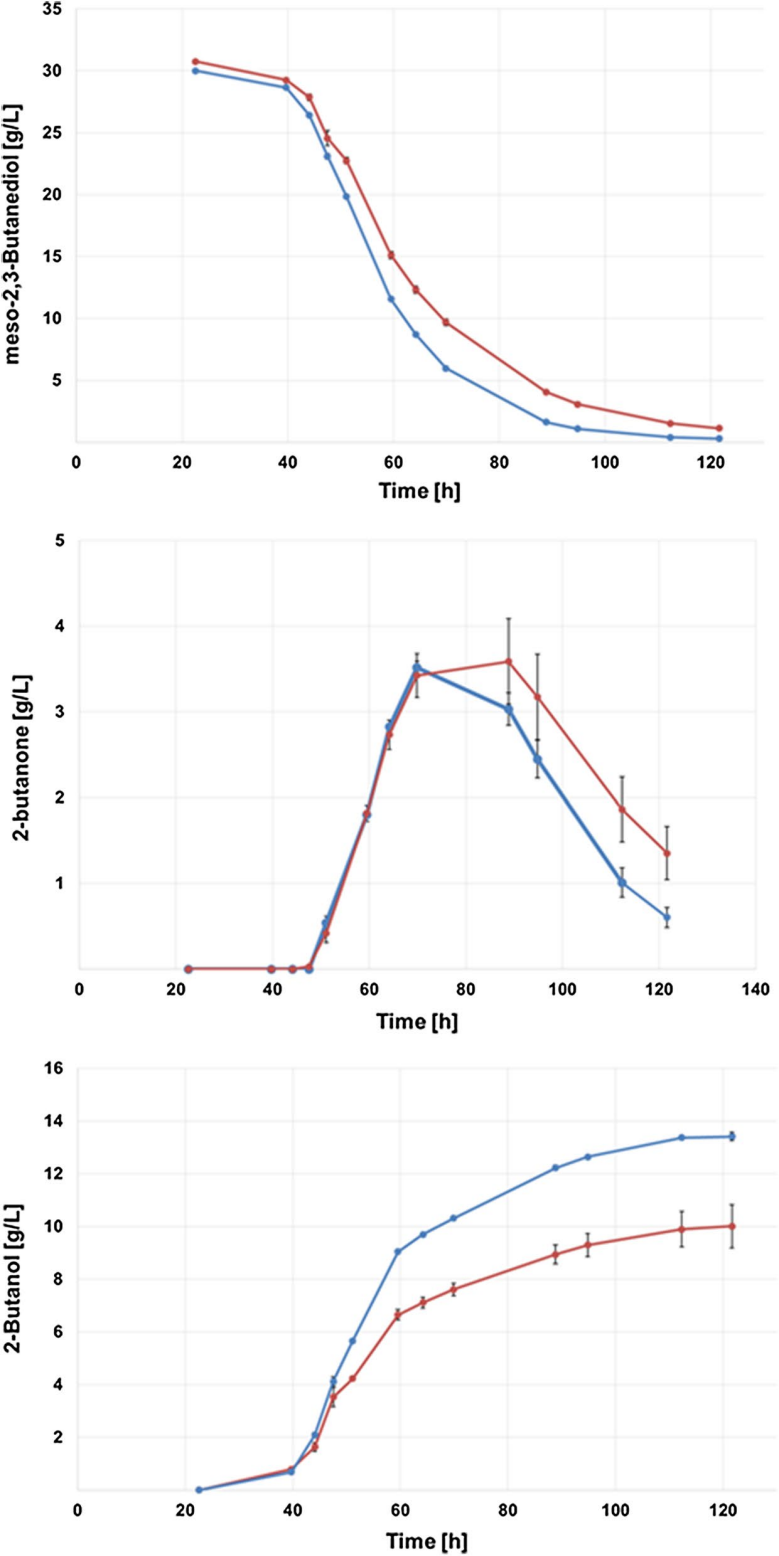
Comparison of 2-butanol production for the wild-type and the pduQ overexpressing strain. Wild type, closed red circles; pduQ overexpression strain, closed turquoise circles. Error bars represent the standard deviation of three independent replicates

Using the overexpression strain, the final 2-butanol titer was increased by 34% reaching a maximum of 13.4 g/L after 112 h of cultivation (Fig. 5). Furthermore, the overall yield on glucose increased to 0.10 g/g. Interestingly, the formation of 2-butanone was still observed and reached still a maximum of around 3.5 g/L which is very similar to the wild-type strain (Fig. 6). However, conversion of 2-butanone was faster and more efficient in the pduQ overexpression strain, as 2-butanone was used up after 121 h (Fig. 6). At the same time point, still 0.3 g/L of 2-butanone was found in the fermentation broth of the wild-type strain. Interestingly, a slightly lower concentration of lactate and an increased acetate concentration were observed (Table 1). This may indicate that additional NADH was created by lactate degradation and further used for reduction of meso-2,3-BTD reduction to 2-butanol. The final ethanol concentrations were the same for the engineered and wild-type strain. Overall, the final 2-butanol titer and conversion rate were increased by the overexpression of pduQ.

## Discussion

So far, no efficient microbial production process for 2-butanol has been described. In this study, we propose *L. diolivorans* as a promising production organism, accumulating a 2-butanol concentration of 13.4 g/L.

The high toxicity of butanol is a general problem during the production process. Therefore, tolerance to butanol is an important point to consider for the selection of a microbial cell factory for this compound. Only few data are available about butanol tolerance of other organisms than *Clostridium* spp. However, some reports showed that *Lactobacillus* spp. have a high tolerance to solvents and acids [12, 15, 22], which makes them interesting hosts for such compounds. Small-scale experiments on MRS + Glucose with increasing 2-butanol concentrations showed that wild-type *L. diolivorans* tolerates 2-butanol up to concentrations of about 30 g/L. This is in good accordance with obtained data for butanol tolerance of other *Lactobacillus* spp. [15, 22, 23]. Interestingly, *Clostridia* spp., the typical organisms used for butanol production, tolerate this solvent only until concentrations of 20 g/L [2, 23]. Typical hosts for metabolic engineering, such as *E. coli* or *S. cerevisiae* already have severe growth problems at butanol concentrations of around 10–15 g/L [15]. This points to the potential of *L. diolivorans* as a cell factory for 2-butanol production.

The metabolic pathway used by *L. diolivorans* for the formation of 2-butanol is a rather simple two-step metabolic pathway. On the contrary, the production of 1-butanol with *Clostridium* spp. underlies complex regulatory mechanisms switching from acidogenesis to solventogenesis [2].

The aforementioned metabolic pathway is widespread among lactic acid bacteria. For example, *Lactobacillus brevis* was also identified to be capable of 2-butanol production. Interestingly, Speranza et al. showed that the glycerol dehydratase of *L. brevis* stereospecifically converts only the meso-form of 2,3-BTD into 2-butanone [17, 21]. The same specificity for the meso-form of 2,3-BTD was also found for the glycerol dehydratase of *L. diolivorans*. *Lactobacillus* strains can produce 2,3-BTD, but product titers are rather low and furthermore, a racemic mixture of all three isoforms is produced. Therefore, *S. marcescens,* a well-known production host known for high yield and stereospecific production of meso-2,3-BTD from glucose, was used for the two-stage production of 2-butanol.

The achieved 2-butanol titer of 13.4 g/L by applying the developed two-step co-cultivation process is around 30 times higher than the so far reported 2-butanol titer in literature (Table 2). In another study, 42 *Lactobacillus* isolates were screened for 2-butanol production from 2,3-BTD, out of which two isolates of *L. brevis* performed best (Table 2). The maximum titer reached was 0.8 g/L of 2-butanol from 3 g/L of meso-2,3-BTD after 170 h of cultivation. Interestingly, 2-butanol production was only observed, when *L. brevis* was cultivated on defined medium. The authors explained the observed result by repression of genes needed for conversion of meso-2,3-BTD due to cultivation on rich media, such as MRS medium [21]. The engineering of *S. cerevisiae* for 2-butanol by introducing the same two-step metabolic pathway, also used by *Lactobacillus* strains, led to a maximum titer of 4 mg/L 2-butanol and 2 mg/L of 2-butanone [24].

**Table 2:** Summary of butanol producing strains

Strain	Product	wt/engin.	Genetic engineering/processes engineering	Titer (g L-1)	Yield (g/g-1)	References
*Lactobacillus diolivorans*	2-Butanol	wt	–	10.0	0.06	This study
*Lactobacillus diolivorans*	2-Butanol	eng.	Overexpression of PduQ	13.4	0.10	This study
*Lactobacillus brevis*	2-Butanol	wt	–	0.88	–	[21]
*Lactobacillus buchneri*	2-Butanol	wt	–	0.04	–	[21]
*Saccharomyces cerevisiae*	2-Butanol	eng.	Overexpression PduCDEGH and ADH	0.01	–	[24]
*Clostridium acetobutylicum*	1-Butanol	wt.	–	11.8	0.18	[2]
*Clostridium acetobutylicum*	1-Butanol	eng.	Knockout of buk and pta	18.9	0.29	[2]
*Clostridium acetobutylicum*	1-Butanol	eng.	Adaptive mutagenesis	20.3	0.23	[4]

*wt* wild type, *eng.* engineered strain

The 2-butanol titers achieved with *L. diolivorans* as production host are comparable to 1-butanol titers achieved by wild-type *C. acetobutylicum*, which are usually around 12 g/L (Table 2). The strategy to improve 2-butanol titers by overexpressing the endogenous primary alcohol dehydrogenase pduQ was successful. Interestingly, 2-butanone formation was still observed and around 3.7 g/L was reached, which is the same concentration also reached for the wild-type strain. In both cases, the accumulated 2-butanone vanished until the end of the fermentation.

A reason for the observed phenomenon can be that pduQ is a primary alcohol dehydrogenase, whereas the reduction of 2-butanone to 2-butanol requires a secondary alcohol dehydrogenase. Therefore, kinetics of the 2-butanone reduction is rather slow as this is not the favoured substrate of pduQ, but overexpression of pduQ still leads to an increase in 2-butanol titers. An approach for further increasing the efficiency of 2-butanol formation will be the overexpression of a heterologous secondary alcohol dehydrogenase. For *S. cerevisiae,* it has been already shown that the overexpression of a secondary alcohol dehydrogenase in combination with the postulated pathway (Fig. 1) allows efficient 2-butanol production.

The theoretical yield for the conversion of 2-butanol from meso-2,3-BTD is 0.82 g/g. For the performed co-cultivation of the wild-type strain, we only reached 0.37 g/g and for the pduQ overexpression strain 0.49 g/g. The difference between the theoretical and observed yield was unexpected, as there are no side products of 2,3-BTD conversion known. Furthermore, we do not find any unidentified peaks in the chromatograms of the supernatants. Therefore, only the degradation of the product or loss of substrate and/or product via the gas phase explains the observed difference in yield. 2-butanone and 2-butanol are volatile substances and evaporation from the fermentation broth is a major problem in industrial processes. It is, therefore, very likely that the constant nitrogen gassing, assuring anaerobic conditions throughout the entire cultivation, causes such an evaporation of 2-butanol and/or 2-butanone and explains the lower observed product yield from meso-2,3-BTD. 2-butanol titers and yields could be further increased by avoiding nitrogen gassing or the implementation of a more efficient cooling trap for the off gas than we actually have. Another point to consider for designing an economically efficient process is the optimization of the cultivation medium. In this study, all performed cultivation steps were done with complex MRS medium to avoid nutrient limitation and allow the best possible conditions for the conversion of meso-2,3-BTD into 2-butanol by *L. diolivorans*. The complex components of the MRS medium, such as casein peptone, meat extract and yeast extract, contribute mainly to the high costs of the MRS medium. A goal for further media optimization can be the reduction or omitting of the complex components.

Another strategy to decrease media costs is the use of a chemically defined medium. For *L. brevis,* it was already shown that conversion of meso-2,3-BTD into 2-butanol is possible on such defined media [21].

## Conclusion

In this study, we identified *L. diolivorans* as potential host for the production of 2-butanol from meso-2,3-BTD during anaerobic glucose fermentation. Up to 10 g/L of 2-butanol was produced by wild-type *L. diolivorans* during a two-step cultivation process with *S. marcescens*. Using an engineered strain of *L. diolivorans*, overexpressing the endogenous alcohol dehydrogenase pduQ, 2-butanol concentrations were further increased to 13.4 g/L. To our knowledge, this is the highest titre described for microbial 2-butanol production so far. The obtained 2-butanol concentrations are in the range of 1-butanol concentrations typically reached by wild-type *Clostridia* strains, which are considered as the best natural producers for butanol.

As *L. diolivorans* is able to tolerate higher 2-butanol concentrations, a further increase in final titers is possible. To reach higher titers, further processes engineering is needed, as evaporation of 2-butanol and the intermediate 2-butanone is a challenge throughout the cultivations and lowers 2-butanol concentrations and yields.

## Materials and methods

### Strains

The *Lactobacillus diolivorans* strains used in this study were LMG 19667 wild type and LMG 19668+pSHM+PDO-DH(NADPH). The engineered strain LMG 19668+pSHM+PDO-DH (pduQ) possesses a overexpression of the endogenous 1,3-propanediol oxidoreductase (PDO-DH) [20]. For bioreactor cultivations, *Serratia marcescens* DSMZ 14187 was used.

Cells were maintained at − 80 °C in culture medium supplemented with 10% (v/v) glycerol.

### 2-Butanol toxicity test

An overnight culture of *L. diolivorans* LMG 19667 in the exponential growth phase was used for inoculation of 2 mL MRS media with increasing concentrations of 2-butanol, ranging from 0 to 60 g/L. The initial OD_600_ for all cultivations was 0.1. The inoculated cultures were incubated at 30 °C and 150 rpm in an anaerobic jar on a rotary shaker for 72 h. The toxicity of 2-butanol was assessed via measurement of OD_600_ after 72 h of incubation.

### Co-cultivation of *S. marcescens* and *L. diolivorans*

Co-cocultivation of *S. marcescens* and *L. diolivorans* was realised in a two-step process. In the first step, a batch cultivation of *S. marcescens* was performed. Therefore, *S. marcescens* batch medium was inoculated to an OD_600_ of 0.2 with an overnight culture. The overnight culture of *S. marcescens* was grown on LB medium at 30 °C and 180 rpm on a shaker.

The co-cultivation was performed in a DASGIP ^®^ parallel bioreactor systems (Eppendorf International). For the bioreactor cultivation, in the first step, the stirrer speed and temperature were set to 400 rpm and 30 °C. The pH was kept constant at 7.0 via addition of 5 M NaOH or 1 M H_3_PO_4_. Batch cultivations were carried out under microaerophilic conditions by gassing with 27 sL/h of air (0.75 vvm). After approximately 19 h, all glucose was depleted and a heat inactivation at 60 °C for 30 min was done and afterwards the medium was cooled again to 30 °C.

Following the cooling phase and addition of 5 times concentrated MRS batch medium to ensure proper growth conditions for *L. diolivorans*, the second step was initiated. In the second step, a *L. diolivorans* overnight culture was used to inoculate the MRS batch medium with an OD_600_ of 0.1. Additionally, for the LMG 19668+pSHM+PDO-DH (pduQ), erythromycin (10 µg/ml) was added to the batch medium to allow stable expression of the gene. The overnight culture for both strains was done on MRS medium with a pH of 5.7. For the engineered strain LMG 19668+pSHM+PDO-DH (pduQ), again erythromycin (10 µg/ml) was added to MRS medium. The overnight culture was incubated at 30 °C and 150 rpm for approximately 20 h. For the bioreactor cultivation stirrer speed was set to 400 rpm and the temperature was set to 30 °C. The pH was kept constant at pH 5.7 via addition of 12.5% NH_3_. The bioreactor cultivation during the second step was carried out under anaerobic condition by gassing with 2 sL/h of nitrogen. For the wild-type and pduQ overexpression strain, three independent replicates were performed.

LB medium contained per litre: 10 g soy peptone, 5 g yeast extract, 5 g NaCl.

*Serratia marcescens* batch medium contained per litre: 33.36 g yeast extract, 11.39 g tri-sodiumcitrate·2H_2_0, 4 g NaAc, 1 g NH_4_(HPO_4_)_2_, 0.62 g MgCl_2_·7H_2_O, 0.11 g MnSO_4_·H_2_O and 99 g glucose·H_2_0 [23].

MRS batch medium contained per litre: 10 g casein peptone, 10 g meat extract, 5 g yeast extract, 1 g tween 80, 2 g K_2_HPO_4_, 5 g NaAc, 2.60 g tri-sodiumcitrate·2H_2_0, 1.17 g NH_4_(HPO_4_)_2_, 0.20 g MgCl_2_·7H_2_O, 0.05 g MnSO_4_·H_2_O, 5 mg vitamine B12 and 33 g glucose·H_2_0.

MRS medium contained per litre: 10 g casein peptone, 10 g meat extract, 5 g yeast extract, 1 g tween 80, 2 g K_2_HPO_4_, 5 g NaAc, 2.60 g tri-sodiumcitrate·2H_2_0, 1.17 g NH_4_(HPO_4_)_2_, 0.20 g MgCl_2_·7H_2_O, 0.05 g MnSO_4_·H_2_O and 22 g glucose·H_2_0.

### HPLC analysis

The concentrations of metabolites (glucose, lactate, acetate, ethanol, meso-2,3-BTD, 2-butanone, 2-butanol) were determined by HPLC (Shimadzu, Korneuburg Austria) equipped with an Aminex HPX-87H column (300 × 7.8 mm, Biorad), which was operated at a temperature of 60 °C and a flow of 0.6 ml/min. As a mobile phase, 4 mM H_2_SO_4_ was used. The samples and standards for HPLC analysis were prepared by mixing 900 µL of sample/standard with 100 µL 40 mM H_2_SO_4_. Subsequently, samples and standards were filtered. For the detection of metabolites, a refraction index detector (RID-10A, Shimadzu, Korneuburg Austria) was used.

## Data Availability

All data generated or analysed during this study are included in this published article.

## Abbreviations

meso-2,3-BTD: meso-2,3-butanediol
ABE: acetone–butanol–ethanol
2,3-BTD: 2,3-butanediol
BMC: bacterial microcompartment
1,3-PDO: 1,3-propanediol
3-HPA: 3-hydroxypropionaldehyde
